# The log odds of positive neck lymph nodes is a superior lymph node predictor for overall survival in head and neck cancer: a population-based analysis in Germany

**DOI:** 10.1007/s00405-021-07176-8

**Published:** 2021-11-22

**Authors:** Mussab Kouka, Elisa Al-Ahmar, Jens Büntzel, Holger Kaftan, Daniel Böger, Andreas Müller, Stefan Schultze-Mosgau, Thomas Ernst, Orlando Guntinas-Lichius

**Affiliations:** 1 Department of Otorhinolaryngology, Jena University Hospital, Am Klinikum 1, 07747 Jena, Germany; 2 Department of Otorhinolaryngology, Suedharzklinikum Nordhausen, Nordhausen, Germany; 3 Department of Otorhinolaryngology, Helios-Klinikum Erfurt, Erfurt, Germany; 4 Department of Otorhinolaryngology, SRH Zentralklinikum Suhl, Suhl, Germany; 5 Department of Otorhinolaryngology, SRH Wald-Klinikum Gera, Gera, Germany; 6 Department of Oromaxillofacial Surgery and Plastic Surgery, Jena University Hospital, Jena, Germany; 7 University Tumor Center, Jena University Hospital, Jena, Germany

**Keywords:** Head and neck cancer, Tumor staging, Cancer registry, Cervical lymph node metastases, Lymph node classifications, Survival

## Abstract

**Background:**

This population-based study investigated the influence of different lymph node (LN) classifications on overall survival (OS) in head and neck cancer (HNC).

**Methods:**

401 patients (median age: 57 years; 47% stage IV) of the Thuringian cancer registries with diagnosis of a primary HNC receiving a neck dissection (ND) in 2009 and 2010 were included. OS was assessed in relation to total number of LN removed, number of positive LN, LN ratio, and log odds of positive LN (LODDS).

**Results:**

Mean number of LODDS was 0–0.96 ± 0.57. When limiting the multivariate analysis to TNM stage, only the UICC staging (stage IV: HR 9.218; 95% CI 2.721–31.224; *p* < 0.001) and LODDS >  – 1.0 (HR 2.120; 95% CI 1.129–3.982; *p* = 0.019) were independently associated with lower OS.

**Conclusion:**

LODDS was an independent and superior predictor for OS in HNC in a population-based setting with representative real-life data.

**Supplementary Information:**

The online version contains supplementary material available at 10.1007/s00405-021-07176-8.

## Introduction

Head and neck cancer (HNC) is the 7th most common cancer worldwide [1]. 12,920 new cases in men and 4,881 in women were reported for Germany for 2015–2016 [2]. Optimal tumor staging is essential for classification, treatment planning and prognosis. Staging is performed according to the TNM (tumor, node, metastasis) classification and the UICC (Union for International Cancer Control) system [3]. Curative and elective neck dissection (ND) as treatment of cervical lymph node metastases play a central role in the therapy of HNC [4–6]. To make correct prognostic statements and to establish an optimal therapeutic concept, the quality of the histopathological findings of the yielded lymph nodes is extremely important. This pN classification is highly dependent on the total number of lymph nodes removed (TNOD) and histopathological specimen in particular [7]. Therefore, additional classifications have been investigated in recent years regarding to their prognostic significance. Roberts et al. identified the number of positive lymph nodes (PNOD) as a valid prognostic classification [8]. In other studies, lymph node ratio (LNR), which is the ratio between PNOD and TNOD, is shown as a superior and independent prognostic classification [9, 10]. There is a strong dependence on the TNOD, which may be influenced by the extent of ND on one hand and accuracy of histopathological findings on the other hand [11]. Recent studies investigated the impact of a new lymph node classification scheme on OS of tumor patients: the log odds of positive lymph nodes (LODDS). LODDS is defined as the logarithm of the ratio between PNOD and negative lymph nodes [7, 12]. LODDS is already used as a prognostic factor in breast carcinoma, gastric cancer, pancreatic cancer and colorectal carcinoma [13, 14]. There are only few hospital-based studies and one multi-institution study so far, which considered the influence of LODDS on OS in HNC patients. In these studies, LODDS has been demonstrated as a prognostic classification superior than others such as pN and LNR [7, 12, 15, 16]. LODDS was able to discriminate patients without positive lymph nodes, few nodes or insufficient nodes retrieved [12].

This study investigates the influence of these extended lymph node classifications on OS for the first time on a population basis. In addition, the influences of patient and tumor parameters as well as aspects of ND on OS were analyzed.

## Methods

### Ethical considerations

The Ethics Committee of the Jena University Hospital approved the study (IRB No. 3204-07/11). The Ethics Committee waived the requirement for informed consent of the patients because the study had a non-interventional retrospective design and all data were analyzed anonymously.

### Patients

This population-based cohort study was established on patients who were diagnosed with a primary HNC in the period from January 2009 to December 2010 and were registered in the five Thuringian cancer registries (Erfurt, Gera,,Jena, Nordhausen, Suhl, and Weimar) covering > 98% of all new HNC patients [17]. These 2 years were selected to allow adequate follow-up. Thuringia is a federal state in Germany and involves a population of about 2.1 million people. In total, 709 new cases of head and neck cancer were registered, i.e., the exact incidence in 2009–2010 was 31.6 new cases per 100,000 habitants. All patients without surgery treatment which included a ND, patients with skin cancer or who had metastasis of other entities in the head and neck were excluded. 401 patients build the final dataset. In addition to the cancer registry data, cases were divided according to the International Classification of Disease for Oncology (ICD-O-3) into lip, oral cavity, nasopharynx, oropharynx, hypopharynx, larynx, salivary glands and nose/paranasal sinus [18]. Furthermore, all pathological stages of the primary cancer were recorded using the TNM-classification, 7th edition [3]: tumor size T, regional cervical lymph node status N, distant metastases M, resection status R, grading G, lymph vessel invasion L, venous invasion V, and extracapsular spreading ECS. In a few cases, the primary head and neck tumor was not resected, but a ND was performed. Since no histopathologically confirmed tumor size was available in these cases, the clinical tumor size cT was integrated into the value labeling of the variables and used for these cases. In addition to the cancer registry data, all necessary information on the ND could be obtained from the surgical and histopathological reports from the patients’ charts.

### Lymph node classification

Lymph node ratio (LNR) was determined as the quotient from the total number of positive lymph nodes (PNOD) and the total number of lymph nodes removed (TNOD) [15]. The natural logarithm of the quotient of PNOD and the number of negative lymph nodes is called LODDS (= log odds of positive lymph nodes) and is calculated as follows: log ((PNOD + 0.5) / (TNOD—PNOD + 0.5)). The value 0.5 was added to both PNOD (= positive number of lymph nodes) and TNOD (= total number of lymph nodes) to avoid a numerical singularity [15].

### Statistical analysis

Descriptive statistical analyses were performed using SPSS Statistics Version 24 (IBM Deutschland GmbH, 71,139 Ehningen, Germany). Nominal parameters, such as patient characteristics, ND and tumor characteristics were analyzed with absolute and relative frequency calculation. Metric data were evaluated by calculating the median and the range as well as the mean and the standard deviation. The influence of all variables on OS was analyzed using the log-rank test. The Kaplan–Meier method was chosen to graphically represent the significant factors influencing OS. A significance level of p = 0.05 was chosen. Variables with *p* < 0.05 were rated as statistically significant. The time periods to be analyzed were 2-year and 5-year survival rates. Multivariable analyses using Cox proportional hazard ratio (HR) with corresponding 95% confidence interval (CI) were performed to verify an independent influence of all significant factors influencing OS in the univariate analysis. A significance level of *p* = 0.05 was set.

## Results

### Patient’s characteristics and tumor characteristics

In total, 401 patients (321 men; 80%) were included, i.e., the ND rate in Thuringia for HNC in 2009–2010 was 17.9 per 100,000 habitants. The median age at diagnosis was 57 years (range 23–86 years). As shown in Table 1, the largest proportion of all malignancies was represented by oral cavity carcinoma (40%). Furthermore, oropharyngeal carcinoma represented a frequent tumor location (21%). Squamous cell carcinomas accounted for 92% of the primary tumors (Table 2). One third of the patients had an advanced T classification (pT3/T4: 35%). Regarding the resection status of the primary tumors, R0 resection was achieved in 82% of patients. R1 resection occurred in 8% and R2 resection in 2% of cases. In 19 patients, no surgical therapy of the primary tumor was performed. At the time of diagnosis, almost half of the patient collective (47%) was already in UICC stage IV. The distribution of the other half of the collective was evenly divided between UICC stages I, II, and III in 16% each. Selective ND was predominantly performed in unilateral operations (81%). Only 33 out of 176 unilateral operations were modified radical or radical ND (19%). In the case of bilateral operations, in two third of the cases (66%) selective ND and in only on third of the cases (34%) modified radical or radical ND was performed on the side of the tumor.

**Table 1:** Patients’ characteristics

Parameter	Frequency (N)	%
Gender		
Male	321	80
Female	80	20
Localization		
Cavity of the mouth	159	40
Oropharynx	83	21
Hypopharynx	49	12
Larynx	55	14
Salivary glands	31	8
Lip	9	2
Nasopharynx	5	1
Nose and paranasal sinus	5	1
Middle ear	1	< 1
CUP	4	1
Neck dissection		
Unilateral	176	42
Bilateral	247	58
Type of neck dissection (unilateral operation)		
Selective	143	81
Modified radical or radical	33	19
Type of neck dissection (bilateral operation)		
Selective (ipsilateral)	163	66
Modified radical or radical (ipsilateral)	84	34
Selective (contralateral)	217	88
Modified radical or radical (contralateral)	30	12
Recurrence		
No	115	29
Yes	286	71
Death		
No	295	74
Yes	106	26
Year of diagnosis		
2009	211	53
2010	190	47
Thuringian tumor registry		
Jena	148	37
Erfurt	134	33
Gera	44	11
Nordhausen	30	8
Suhl	45	11

	Mean ± SD	Median, Range
Age (years)	58.7 ± 10.4	57, 23–86
Follow-up (months)	48 ± 32.1	51, 0–115
Follow-up of patients alive (months)	58 ± 30.4	62, 0–115

^*^CUP carcinoma of unknown primary, *SD *standard deviation

**Table 2:** Histopathology and lymph node characteristics

Parameter	Frequency (N)	%
pT classification		
T0/Tis	6	1
T1	102	25
T2	126	31
T3	74	18
T4	65	16
Tx	28	7
pN classification		
N0	189	47
N1	50	13
N2	154	38
N3	4	1
Nx	4	1
M classification		
M0	378	94
M1	9	2
Mx	14	3
Stage (AJCC 7th edition 2010)		
I	5	1
II	63	16
III	66	16
IV	189	47
Unstaged	14	3
Tumor margins		
R0	327	82
R +	42	10
Rx	8	8
No primary tumor excision	19	5
Missing	5	1
Tumor differentiation		
G1/2	260	65
G3	111	28
Unknown	30	7
Lymphovascular invasion		
L0	186	46
L1	77	19
Unknown	138	34
Venous invasion		
V0	238	59
V1	11	3
Unknown	152	38
Extracapsular spread		
ECS-	66	16
ECS +	55	14
Unknown	90	22
N0/Nx	190	47
Histology		
Squamous cell carcinoma	368	92
Adenocarcinoma	7	2
Other carcinoma	26	6

	Mean ± SD	Median, Range
Type of neck dissection (unilateral operation)		
Lymph nodes resected (TNOD)	13.6 ± 10.4	11, 0–63
Positive lymph nodes (PNOD)	1.4 ± 2.7	0, 0–16
Lymph node ratio (LNR)	0.16 ± 0.27	0, 0–1
Log odds of positive lymph nodes (LODDS)	– 0.96 ± 0.57	– 1.11, – 2 to 0.66
Type of neck dissection (bilateral operation)		
Lymph nodes resected (TNOD)	29.5 ± 16.3	27, 0–84
Positive lymph nodes (PNOD)	2.2 ± 4.1	1, 0–35
Lymph node ratio (LNR)	0.09 ± 0.15	0.03, 0–1
Log odds of positive lymph nodes (LODDS)	– 1.2 ± 0.57	– 1.24, – 2.14 to 1.04

^*^AJCC American joint committee on cancer, *TNOD *total number of lymph node, *PNOD *number of positive lymph node, *LNR *lymph node ratio, *LODDS *log odds of positive lymph node, *ECS *extracapsular spread, *SD *standard deviation

### Cervical lymph node status and classifications

Overall, regional lymph node metastasis was histologically proven in more than half of all patients (pN + ; N = 208; 52%). Most of these patients (74%) had a pN2 lymph node status. In addition, extracapsular spreading (ECS) of lymph node metastases was detected in 66 of 208 cases (32%). In 90 of 208 cases (43%), no statement was made regarding ECS. In all unilateral ND, a mean number of 13.6 ± 10.4 lymph nodes were yielded. Of these, a mean number of 1.4 ± 2.7 lymph nodes were positive (Median 0, range = 0–16). The mean LNR was 0.16 ± 0.27 (Median 0, range = 0–1). The median LODDS was -0.96 ± 0.57 (Median -1.11, range =  – 2 to 0.66). In all ND performed bilaterally, a mean number of 29.5 ± 16.3 lymph nodes were yielded. Of these, a mean number of 2.2 ± 4.1 lymph nodes were positive (Median 1, range = 0–35). The mean LNR was 0.09 ± 0.15 (Median 0.03, range = 0–1). The mean LODDS was  – 1.2 ± 0.57 (Median -1.24, range =  – 2.14 to 1.04).

### Overall survival

**Fig. 1:**
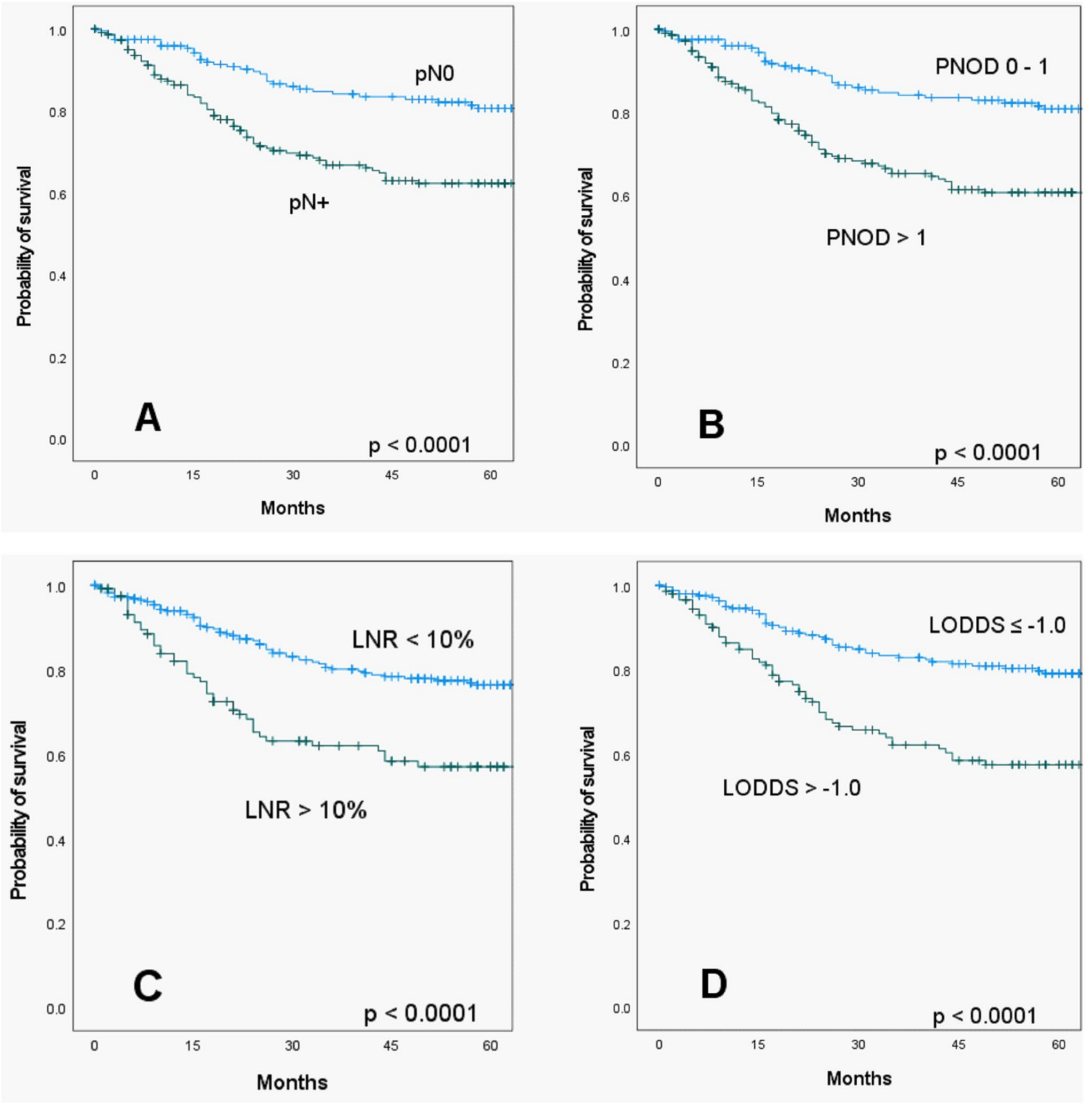
Kaplan–Meier curves of overall survival according to pN status (**A**), number of positive lymph nodes (PNOD) (**B**), lymph node ratio (LNR) (**C**), and log odds of positive lymph nodes (LODDS) (**D**)

Median follow-up was 51 months (range: 0–115) and median follow-up for patients alive was 62 months (range (0–115). The univariable analyses showed (Supplementary Table S1) that higher age was associated with lower survival probability (*p* = 0.020). Tumor parameters pT, pN and M showed significant influence on OS. Patients with a T3/T4 tumor had a significantly worse 5-year OS rate (5-year OS = 55.7%; *p* < 0.001) than patients with a T1/T2 tumor (5-year OS = 78.5%). Increasing lymph node involvement, the presence of distant metastases and advanced stage of the UICC tumor classification resulted in a lower OS (all *p* < 0.05). Incomplete resection (R1) results in worse outcome (*p* < 0.0001). Existing lymphatic vessel invasion as well as venous invasion also were negative prognostic factors for OS (both *p* < 0.001). Oropharyngeal carcinoma was the only tumor entity to show significantly better OS than the other localizations (*p* = 0.040). The presence of more than one affected lymph node was associated with a reduction in OS (5-year OS = 59.6%) compared to PNOD = 0–1 (5-year OS = 77.2%; *p* < 0.001; Fig. 1). A LNR between 0 and 10% showed a 2-year OS of 86.5%. A LNR of > 10% reduced the 2-year OS by 20.8% (*p* =  < 0.001). The 5-year OS rate of LODDS ≤  – 1.0 was 79.0%. LODDS >  – 1.0 resulted in a reduced 5-year OS of 57.2% (*p* < 0.001).

In a multivariable analysis of all variables influencing OS, (model I, Table 3), the only remaining independent risk factors were age > 57 years, a pT4 tumor, and incomplete tumor resection (R1). Accordingly, patients older than 57 years had a 2.21-fold increased hazard of death (*p* = 0.007). Patients who had a pT4 tumor, had a 3.38-fold increased hazard of death (*p* = 0.045). The hazard of death was increased by 3.52 for patients with incomplete resections. To focus on the role of the lymph node status, only the UICC stage, the number of positive lymph nodes (PNOD) and the lymph node indices (LNR and LODDS) were included in a second multivariable model (model II, Table 3). A UICC stage II had a 5.381-fold increased hazard of death (*p* = 0.008). At a UICC stage III, hazard of death increased (HR 4.312; 95% CI 1.234–15.067, *p* = 0.022) and at the highest tumor stage, UICC stage IV, there was an 9.218-fold increased hazard of death (HR 9.218; 95% CI 2.721–31.224; *p* < 0.001). The number of positive lymph nodes as well as the lymph node ratio showed no independent influence on OS. In contrast, the hazard of death increased for LODDS >  – 1.0 (HR 2.120; 95% CI 1.129–3.982; *p* = 0.019).

**Table 3:** Multivariable analysis of risk factors for head and neck cancer overall survival

Factor		HR*	Lower 95%CI	Upper 95%CI	p*
Model I					
Age	≤ 57 years	1	Reference		
	> 57 years	2.21	1.240	3.938	**0.007**
Gender	Female	1	Reference		
	Male				
pT	T1	1	Reference		
	T2	1.242	0.397	3.887	0.710
	T3	1.858	0.595	5.796	0.286
	T4	3.378	1.027	11.112	**0.045**
pN	N0	1	Reference		
	N1	1.005	0.341	2.962	0.992
	N2	1.341	0.347	5.184	0.671
	N3				
M	M0	1			
	M1	1.995	0.396	10.048	0.402
R	R0	1			
	R1	3.523	1.492	8.317	**0.004**
Stage	I	1	Reference		
	II	4.004	0.599	26.772	0.152
	III	3.220	0.481	21.547	0.228
	IV	3.700	0.503	27.200	0.199
L	L0	1	Reference		
	L1	1.039	0.533	2.027	0.910
V	V0	1	Reference		
	V1	1.779	0.684	4.625	0.237
Tumor localization	Other localization	1	Reference		
	Oropharynx	0.460	0.194	1.093	0.079
Ipsilateral neck dissection	Modified radical/radical	1	Reference		
	Selective	0.953	0.531	1.712	0.873
PNOD	0–1	1	Reference		
	> 1	1.112	0.368	3.353	0.851
LNR	0–10%	1	Reference		
	> 10%	0.606	0.230	1.602	0.313
LODDS	≤ −1,0	1	Reference		
	> −1,0	2.166	0.860	5.456	0.101
Model II					
Stage	I	1	Reference		
	II	5.381	1.557	18.589	**0.008**
	III	4.312	1.234	15.067	**0.022**
	IV	9.218	2.721	31.224	** < 0.001**
PNOD	0–1	1	Reference		
	> 1	0.677	0.362	1.266	0.222
LNR	0–10%	1	Reference		
	> 10%	0.808	0.420	1.552	0.522
LODDS	≤ −1,0	1	Reference		
	> −1,0	2.120	1.129	3.982	**0.019**

^*^Statistically significant difference compared to reference (*p* < 0.05), *HR *hazard ratio, *CI *confidence interval, *TNOD *total number of lymph nodes, *PNOD *number of positive lymph node, *LNR *lymph node ratio, *LODDS *log odds of positive lymph nodes

## Discussion

In this population-based study, different lymph node classifications were analyzed for OS in all new HNC treated in a federal state of Germany. LODDS > -1.0 was validated as the only independent significant lymph node predicator for lower OS. PNOD and LNR showed no independent influence on OS. LODDS has been shown in hospital-based data as an independent predictor of OS in other carcinoma types [7, 13, 14, 19, 20]. In addition, recent studies have investigated the impact of LODDS on HNC tumors. Yildiz et al. showed in a retrospective single-institution study of 225 patients that LODDS was an independent prognostic factor. LODDS >  – 1.0 was a predictor for lower OS [15]. In a multi-institution retrospective analysis of 3958 cases of oral cancer based on the Surveillance, Epidemiology and End Results (SEER) database, LODDS was shown to be a more accurate predictor of the 5-year disease specific survival than other lymph node classifications [21]. In this SEER study, all patients with fewer than ten lymph nodes yielded were excluded because this was defined as an inadequate ND. This makes it difficult to make final conclusions about the quality of LODDS especially in the case of few yielded lymph nodes. Safi et al. demonstrated in a retrospective single-institution study of 499 patients with oral cancer that LODDS is a better predictor of regional recurrence than LNR or PNOD [22]. In contrast, Subramaniam et al. showed different results in a retrospective single-institution analysis of 643 patients with oral cancer. The PNOD and LNR were the best predictor for OS and disease free survival [23]. Subramaniam divided LODDS in three risk subcategories. Patient with the lowest subcategory (LODDS <  – 1.68) were not included. In their analysis, patients with higher risk subcategories were overrepresented and the cut-off-values for the different risk subcategories for LODDS was subject. Additionally, they suggested that pN and LNR were better in terms of model discrimination [23]. Jin et al. demonstrated in a retrospective single-institution study again only focused on 233 patients with oral cancer that LODDS has the stronger predictive power compared to LNR and PNOD. LODDS was the best lymph node predictor for 10-year disease-specific survival. The cutoff value for a lower disease-specific survival was LODDS >  – 1.491. LODDS can differentiate better than LNR and PNOD, especially when TNOD < 18 or few positive lymph nodes were yielded in inadequately performed ND. In addition, by adding 0.5 to the denominator and numerator, LODDS can differentiate OS of patients without positive lymph nodes [16]. In contrast, Bao et al. do not indicate a predictive advantage of LODDS over PODS with respect to 5-year OS in a prospective study of 706 patients with oral cancer. Furthermore, they showed a nonlinear relationship between OS and the number of negative lymph nodes. OS was reduced and performance of LODDS as lymph node predictor was decreased when more than 40 negative lymph nodes were yielded. It was speculated that the lower OS with increasing lymph node yield was caused by increased postoperative complications caused by the more extensive lymphadenectomy[24]. This kind of nonlinear relation between OS and the number of negative lymph nodes was already reported by Ebrahimi et al. [25]. The study of Bao et al. has some limitations. First of all, it is a single-institution study. Bao et al. divided also LODDS in three subcategories and the cut-off-values for the different subcategories was subject. They did not evaluate other lymph node classification rather than other prognostic values such as the type of ND.

According to the present analysis that included all types of HNC, LODDS may give a better and more accurate indication of OS, especially in patients without positive lymph nodes. The present study is the first population-based analysis of its kind and can accordingly give a very good patient representative of a real-world clinical routine setting. The sample size was relatively large and so differences of head and neck subsites could be analyzed. However, this study has some limitations that need to be addressed. The retrospective analysis cannot guarantee a standardized treatment decision of the different head and neck surgeons during ND. It is well known that surgical standardization increases the quality and number of yielded lymph nodes in a ND [11]. The number of yielded lymph nodes and the decision to perform unilateral or bilateral ND were also not standardized. The average number of lymph nodes removed in unilateral ND in this study was 13.6 lymph nodes. However, a sampling of 18 lymph nodes is recommended. The quality of ND increases with the number of at least 18 lymph nodes [25, 26]. In addition, there is no standardization in the reporting of lymph nodes by pathologists.

## Conclusion

This study provides a first population-based analysis and can give a much better assessment of lymph node parameters than a single-institution study. LODDS seems to be a promising predictor. Confirmation of the results with a larger patient population in a prospective trial and at best also in other countries is needed. The LODDS cutoff values were set by several authors subjectively and have to be standardized and validated. In conclusion, the results of this study demonstrate superiority of the lymph node classification LODDS as a survival predictor for HNC over the commonly used classification LNR and PNOD. However, further research is needed to evaluate the reliability of LODDS in the differentiation in numbers of yielded lymph nodes.

## Supplementary Information

Below is the link to the electronic supplementary material.

Supplementary file1 (DOCX 63 KB)
