# Supplementary material for: The log odds of positive neck lymph nodes is a superior lymph node predictor for overall survival in head and neck cancer: a population-based analysis in Germany
Source: Eur Arch Otorhinolaryngol. 2021 Nov 22;279(7):3587–95. doi: 10.1007/s00405-021-07176-8 (PMC9130179; doi:10.1007/s00405-021-07176-8)
Supplement: Supplementary file 1 — Supplementary file1 (DOCX 63 KB) [file 405_2021_7176_MOESM1_ESM.docx]

**SUPPLEMENTARY TABLES**

**Supplementary Table S1**

| **Supplementary Table S1.** Univariable predictors for overall survival (OS) | | | |
| --- | --- | --- | --- |
| **Parameter** | **2-Year OS (%)** | **5-Year OS (%)** | **p** |
| Gender |  |  | 0.3 |
| Male | 79.4 | 69.7 |  |
| Female | 84.7 | 75.4 |  |
| Age (median 57 years) |  |  | **0.02** |
| ≤ median | 83.2 | 75.2 |  |
| > median | 77.1 | 65.3 |  |
| Localization |  |  | 0.22 |
| Cavity of the mouth | 82.2 | 71.8 |  |
| Oropharynx | 87.6 | 81.7 |  |
| Hypopharynx | 68.1 | 62.6 |  |
| Larynx | 82.4 | 64.5 |  |
| salivary glands | 72.1 | 64.5 |  |
| Lip | 87.5 | 87.5 |  |
| Nasopharynx | 80.0 | 53.3 |  |
| Nose and paranasal sinus | 40.0 | 40.0 |  |
| Middle ear | 0 | 0 |  |
| Localization hypopharynx |  |  | 0.199 |
| Yes | 68.1 | 62.6 |  |
| No | 82.1 | 71.9 |  |
| Localization larynx |  |  | 0.667 |
| Yes | 82.4 | 64.5 |  |
| No | 80.0 | 71.9 |  |
| Localization oropharynx |  |  | **0.040** |
| Yes | 87.6 | 81.7 |  |
| No | 78.4 | 67.8 |  |
| Localization cavity of the mouth |  |  | 0.922 |
| Yes | 82.2 | 71.8 |  |
| No | 79.1 | 70.0 |  |
| Localization salivary glands |  |  | 0.368 |
| Yes | 72.1 | 64.5 |  |
| No | 81.1 | 71.3 |  |
| pT classification |  |  | **<0.0001** |
| T1/T2 | 87.0 | 78.5 |  |
| T3/T4 | 67.4 | 55.7 |  |
| pN classification |  |  | **0.001** |
| pN0 | 89.0 | 79.7 |  |
| pN+ | 74.4 | 63.1 |  |
| M classification |  |  | **0.009** |
| M0 | 82.8 | 71.6 |  |
| M1 | 44.4 | 44.4 |  |
| R classification |  |  | **<0.0001** |
| R0 | 86.7 | 76.0 |  |
| R1 | 45.1 | 33.4 |  |
| Grading |  |  | 0.073 |
| G1 | 91.3 | 88.0 |  |
| G2 | 80.7 | 70.5 |  |
| G3 | 75.8 | 64.1 |  |
| L classification |  |  | **<0.0001** |
| L0 | 87.5 | 79.1 |  |
| L1 | 71.0 | 52.6 |  |
| V classification |  |  | **<0.0001** |
| V0 | 83.8 | 75.0 |  |
| V1 | 40.0 | 30.0 |  |
| Extracapsular spread |  |  | 0.073 |
| ECS- | 81.5 | 66.5 |  |
| ECS+ | 60.4 | 51.4 |  |
| UICC stage |  |  | **<0.0001** |
| I | 96.7 | 96.7 |  |
| II | 88.2 | 75.0 |  |
| III | 88.6 | 73.5 |  |
| IV | 69.2 | 59.5 |  |
| PNOD |  |  | **<0.0001** |
| 0-1 | 86.1 | 77.2 |  |
| >1 | 70.9 | 59.6 |  |
| TNOD |  |  | 0.102 |
| 0-20 | 83.0 | 76.4 |  |
| >20 | 78.7 | 66.1 |  |
| LNR |  |  | **<0.0001** |
| 0-10% | 86.5 | 76.2 |  |
| >10% | 65.7 | 56.8 |  |
| LODDS |  |  | **<0.0001** |
| ≤-1,0 | 87.4 | 79.0 |  |
| >-1,0 | 69.9 | 57.2 |  |
| Neck dissection |  |  | 0.549 |
| Unilateral | 81.6 | 73.0 |  |
| Bilateral | 81.0 | 69.3 |  |
| Type of neck dissection (ipsilateral) |  |  | 0.021 |
| Selective | 82.6 | 73.6 |  |
| Modified radical or radical | 75.9 | 64.9 |  |
| Type of neck dissection (contralateral) |  |  | 0.056 |
| Selective | 80.4 | 69.2 |  |
| Modified radical or radical | 71.6 | 59.7 |  |

TNOD = total number of lymph node; PNOD = number of positive lymph node; LNR = lymph node ratio; LODDS = log odds of positive lymph node; ECS = extracapsular spread
